# Risk factors of hemodialysis catheter dysfunction in patients undergoing continuous renal replacement therapy: a retrospective study

**DOI:** 10.1186/s12882-023-03383-z

**Published:** 2023-11-10

**Authors:** Leerang Lim, Jung Yeon Park, Hannah Lee, Seung-Young Oh, Christine Kang, Ho Geol Ryu

**Affiliations:** 1 Department of Anesthesiology and Pain Medicine, Seoul National University Hospital, Seoul National University College of Medicine, Daehak-Ro 101, Jongno-Gu, Seoul, 03080 Korea; 2 Department of Critical Care Medicine, Seoul National University Hospital, Daehak-Ro 101, Jongno-Gu, Seoul, 03080 Korea; 3 Department of Surgery, Seoul National University Hospital, Seoul National University College of Medicine, Daehak-Ro 101, Jongno-Gu, Seoul, 03080 Korea

**Keywords:** Hemodialysis catheter, Catheter dysfunction, Continuous renal replacement therapy

## Abstract

**Background:**

Continuous renal replacement therapy is a relatively common modality applied to critically ill patients with renal impairment. To maintain stable continuous renal replacement therapy, sufficient blood flow through the circuit is crucial, but catheter dysfunction reduces the blood flow by inadequate pressures within the circuit. Therefore, exploring and modifying the possible risk factors related to catheter dysfunction can help to provide continuous renal replacement therapy with minimal interruption.

**Methods:**

Adult patients who received continuous renal replacement therapy at Seoul National University Hospital between January 2019 and December 2021 were retrospectively analyzed. Patients who received continuous renal replacement therapy via a temporary hemodialysis catheter, inserted at the bedside under ultrasound guidance within 12 h of continuous renal replacement therapy initiation were included.

**Results:**

A total of 507 continuous renal replacement therapy sessions in 457 patients were analyzed. Dialysis catheter dysfunction occurred in 119 sessions (23.5%). Multivariate analysis showed that less prolonged prothrombin time (adjusted OR 0.49, 95% CI, 0.30–0.82, *p* = 0.007) and activated partial thromboplastin time (adjusted OR 1.01, 95% CI, 1.00–1.01, *p* = 0.049) were associated with increased risk of catheter dysfunction. Risk factors of re-catheterization included vascular access to the left jugular and femoral vein.

**Conclusions:**

In critically ill patients undergoing continuous renal replacement therapy, less prolonged prothrombin time was associated with earlier catheter dysfunction. Use of left internal jugular veins and femoral vein were associated with increased risk of re-catheterization compared to the right internal jugular vein.

## Background

Continuous renal replacement therapy (CRRT) is a life-saving modality for hemodynamically unstable critically ill patients requiring renal replacement therapy (RRT) [1–3]. The increasing incidence of acute kidney injury [4] had led to higher utilization of CRRT worldwide [5, 6]. The use of CRRT in Korea has tripled in the past 10 years [7]. For timely initiation and adequate delivery of CRRT, the temporary hemodialysis catheter is the recommended option for vascular access [8].

With a reported mortality of up to 70% in critically ill patients requiring CRRT, along with more comorbidities and higher disease severity [9, 10], adequate delivery of CRRT with minimal interruption is critical for patient receiving CRRT, especially during early period. For stable and consistent application of CRRT, maintaining sufficient blood flow through a patent circuit is important [11]. With dialysis catheter dysfunction, reduced blood flow leads to inadequate pressures within the circuit and circuit clotting [12]. Reduced blood flow and repeated interruption of CRRT delays the correction of electrolyte and metabolic derangement, making it difficult to deliver the intended dose [11–13]. Therefore, exploring and modifying the possible risk factors related to catheter dysfunction may contribute to successful maintenance of vascular access for CRRT with minimal interruption.

Most studies reporting risk factors for catheter dysfunction have evaluated tunneled catheters used for intermittent hemodialysis (iHD) [14–16]. Studies that did investigate catheter dysfunction during CRRT mostly focused on vascular access, comparing access sites or types of catheters [11, 17, 18]. Considering that the blood flow rate during CRRT is significantly slower compared to iHD and that catheter locking with anticoagulants is usually not feasible, factors associated with catheter dysfunction is likely to be different between patients undergoing iHD and CRRT. A systematic review showed that factors associated CRRT filter lifespan included transfusion and severity of illness, in addition to factors related to vascular access [19]. In this study, risk factors associated with dialysis catheter dysfunction within 48 h after initiation of CRRT in critically ill patients were analyzed.

## Methods

This retrospective single center cohort study was approved by the Institutional Review Board of Seoul National University Hospital (IRB No. 2201–020-1286). Written informed consent was waived by the review board due to the retrospective nature of the study.

### Study population and data collection

The electronic medical records of all adult patients (18 years or older) who received CRRT at Seoul National University Hospital between January 2019 and December 2021 were reviewed. Patients who underwent CRRT using hemodialysis catheters inserted at bedside after admission to intensive care units (ICU) within 12 h before initiation of CRRT were included for analysis. Patients who received CRRT using tunneled or non-tunneled catheters which were not inserted in the ICU were excluded. Patients with a properly functioning catheter, but no longer required CRRT within 48 h after initiation were also excluded from the analysis. Patients admitted for active coronavirus infection (COVID-19) were also excluded due to the hypercoagulability associated with the infection [20].

Baseline characteristics including age, sex, comorbidities, medications, and acute physiology and chronic health evaluation (APACHE) II scores at the time of ICU admission were collected. Data relevant to renal replacement therapy such as blood flow rate, intra-circuit anticoagulants, types of vascular access, and the catheter tip location were collected. Catheter tip locations were collected by reviewing x-ray images performed following catheterization and were classified into superior vena cava, right atrium, left innominate vein, iliac vein, and inferior vena cava. The cause of CRRT initiation was evaluated considering the clinical presentations, laboratory results, and imaging tests. Laboratory values including platelet count, prothrombin time – international normalized ratio (PT-INR), activated partial thromboplastin time (aPTT), and high-sensitivity C-reactive protein (hs-CRP) and were also obtained.

### Renal replacement therapy and catheter management

During the study period, the dose, blood flow rate, type of dialysis solution or use of anticoagulant within the circuit were determined by physicians taking care of the patient. Two types of CRRT machines, the Prismaflex® system (Baxter International Inc., Deerfield, IL, USA) and the multiFiltrate® system (Fresenius Medical Care, Bad Homburg vor der Höhe, Germany) was in use. For intra-circuit anticoagulation, nafamostat mesilate was used at a dose of 20 mg/hr to increase filter lifespan without increasing the risk of bleeding [21, 22] at the discretion of the physician. Initiation and discontinuation of CRRT, as well as the decision to convert to iHD, were at the discretion of the attending physician.

Under ultrasonography guidance, 11.5 Fr dual lumen acute dialysis catheters (Medtronic PLC, Dublin, Ireland) were placed at bedside. The site for vascular access and the insertion depth of the catheter was left at the discretion of the physician. In general, 16 cm catheters were used for right internal jugular vein access and 19.5 cm catheters were used for left internal jugular vein or femoral vein access.

### Definition

Catheter dysfunction was defined as the need for catheter handling, including switching between the access and return lumens, catheter repositioning, or re-catheterization within 48 h to maintain adequate pressure and blood flow within the circuit. A CRRT session was defined as the period from the initiation to the termination of CRRT or temporal cessation for re-catheterization. For patient with multiple sessions of CRRT during the period, only the first session of each separate vascular access was included for analysis. Consequently, the maximum number of sessions that could be included was 4 per patient.

### Statistical analysis

Each CRRT session after catheterization was analyzed independently. Data were expressed as mean (standard deviation) or median [interquartile range] for continuous variables, and number (%) for categorical variables. Student’s t-test or Wilcoxon rank-sum test were used for continuous variables and Chi-square test or Fisher’s exact test for categorical variables as appropriate.

To explore risk factors associated with catheter dysfunction, univariate and multivariate generalized estimating equations with robust variance estimator was conducted. Variables with a *p* value of less than 0.1 in the univariate analysis and other relevant variables were included in the multivariate analysis. Considering that the incidence of re-catheterization is typically higher than switching the lumen of the catheter or repositioning the catheter, risk factors for re-catheterization were also evaluated. Timing of catheter dysfunction was compared among vascular access sites using the log-rank test and was expressed as Kaplan–Meier curves.

All statistical analyses and graphics were performed using R 3.6.3 (The R Foundation for Statistical Computing). A *p* value < 0.05 was considered statistically significant.

## Results

**Fig. 1:**
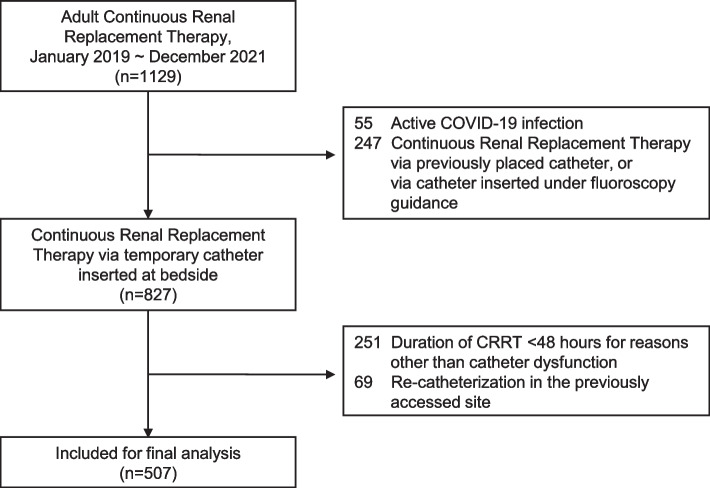
Flow chart of the study

During the study period, 1129 sessions of CRRT were performed in the ICUs, of which 507 sessions of 457 patients were included for analysis (Fig. 1). Catheter dysfunction occurred in 119 sessions among the 507 sessions. There were 82 cases of switch of access-return lumen, 6 cases of catheter reposition, and 31 cases of re-catheterization. Baseline characteristics are shown in Table 1.

**Table 1:** Baseline characteristics according to catheter dysfunction

	No dysfunction (*n* = 388)	Dysfunction (*n* = 119)	Interventions in response to dysfunction		
			Switch of lumen (*n* = 82)	Catheter reposition (*n* = 6)	Re-catheterization (*n* = 31)
Age, yr, median [IQR]	66.0 [57.0–74.0]	69.0 [61.5–77.0]	69.0 [62.3–77.0]	72.5 [41.0–74.8]	68.0 [62.0–77.0]
Sex (F/M)	141/247	48/71	32/50	2/4	14/17
Weight, kg, mean ± SD	63.4 ± 14.1	62.6 ± 12.5	63.5 ± 12.5	59.4 ± 11.4	60.8 ± 12.8
Height, cm, mean ± SD	163.0 ± 8.8	162.1 ± 8.7	162.5 ± 8.6	160.6 ± 10.9	161.5 ± 8.9
Body mass index, kg/m^2^, mean ± SD	23.8 ± 4.5	23.8 ± 4.1	24.0 ± 4.0	22.9 ± 3.4	23.4 ± 4.6
Comorbidities, n (%)					
Hypertension	219 (56%)	71 (60%)	48 (59%)	6 (100%)	17 (55%)
Diabetes mellitus	166 (43%)	52 (44%)	37 (45%)	4 (67%)	11 (35%)
Dyslipidemia	140 (36%)	45 (38%)	33 (40%)	2 (33%)	10 (32%)
Atrial fibrillation	50 (13%)	13 (11%)	8 (9.8%)	0 (0%)	5 (16%)
Myocardial infarction	71 (18%)	16 (13%)	14 (17%)	1 (17%)	1 (3.2%)
Stroke	31 (8.0%)	12 (10%)	12 (15%)	0 (0%)	0 (0%)
Chronic kidney disease	117 (30%)	39 (33%)	28 (34%)	1 (17%)	10 (32%)
Intermittent hemodialysis	65 (17%)	24 (20%)	19 (23%)	1 (17%)	4 (13%)
History of any thrombosis event	41 (11%)	8 (6.7%)	6 (7.3%)	1 (17%)	1 (3.2%)
APACHE II score, median [IQR]	31.0 [25.0–37.0]	33.0 [25.0–39.0]	32.0 [25.0–38.3]	33.0 [30.3–35.5]	33.5 [25.5–39.0]
Medications, n (%)					
Antiplatelet	74 (19%)	17 (14%)	13 (16%)	0 (0%)	4 (13%)
Anticoagulant	76 (20%)	23 (19%)	17 (21%)	2 (33%)	4 (13%)
Vascular access, n (%)					
Catheterized vessel					
Right internal jugular vein	185 (48%)	45 (38%)	37 (45%)	4 (67%)	4 (13%)
Left internal jugular vein	54 (14%)	19 (16%)	11 (13%)	0 (0%)	8 (26%)
Femoral vein	149 (38%)	55 (46%)	34 (41%)	2 (33%)	19 (61%)
Catheter tip location					
Superior vena cava	173 (45%)	49 (41%)	40 (49%)	3 (50%)	6 (19%)
Right atrium	45 (12%)	7 (5.9%)	3 (3.7%)	1 (17%)	3 (10%)
Left innominate vein	21 (5.4%)	9 (7.6%)	6 (7.3%)	0	3 (10%)
Inferior vena cava	9 (2.3%)	2 (1.7%)	2 (2.4%)	0	0
Iliac vein	140 (36%)	52 (44%)	31 (38%)	2 (33%)	19 (61%)
Concurrent catheter in same access	171 (44%)	50 (42%)	40 (82%)	2 (33%)	8 (26%)
Concurrent catheter tip	152 (39%)	45 (38%)	32 (39%)	2 (33%)	11 (35%)
CRRT variables					
Blood flow, L/min, mean ± SD	117.3 ± 23.7	118.0 ± 21.3	116.0 ± 16.7	127.5 ± 40.2	121.5 ± 26.8
Circuit anticoagulation, n (%)	238 (61%)	81 (68%)	55 (67%)	4 (67%)	22 (71%)
Causes of CRRT					
End stage renal disease	72 (19%)	25 (21%)	18 (22%)	1 (17%)	6 (19%)
Sepsis	117 (30%)	44 (37%)	26 (32%)	3 (50%)	15 (48%)
Nephrotoxic kidney injury	32 (8.2%)	9 (7.6%)	5 (6.1%)	0	4 (13%)
Ischemic kidney injury	133 (34%)	34 (29%)	27 (33%)	2 (33%)	5 (16%)
Others	34 (8.8%)	7 (5.9%)	6 (7.3%)	0	1 (3.2%)
Use of vasopressor, n (%)	294 (76%)	87 (73%)	58 (71%)	4 (67%)	25 (81%)
Platelet count, × 10^3^/µL, mean ± SD	97.7 ± 72.2	88.2 ± 57.7	87.6 ± 55.8	110.2 ± 72.8	85.7 ± 60.8
Prothrombin time, INR, mean ± SD	1.8 ± 1.1	1.5 ± 0.6	1.5 ± 0.6	1.7 ± 0.3	1.6 ± 0.7
aPTT, sec, mean ± SD	52.9 ± 29.8	53.3 ± 41.7	55.5 ± 48.7	48.2 ± 24.6	48.6 ± 18.6
hs-CRP level, mg/dL, mean ± SD	12.3 ± 9.8	12.0 ± 9.3	11.6 ± 9.2	11.9 ± 7.7	13.0 ± 9.7

*Abbreviations*: *IQR* interquartile range, *SD* standard deviation, *APACHE* acute physiology and chronic health evaluation, *CRRT* continuous renal replacement therapy, *INR* international normalized ratio, *aPTT* activated partial thromoplastin time, *hs-CRP* high sensitivity C-reactive protein

Patients with catheter dysfunction were significantly older compared to patients without catheter dysfunction (66.0 (57.0–74.0) vs. 69.0 (61.5–77.0), *p* = 0.005). Regarding the vascular access, the right internal jugular vein was most frequently accessed for catheterization (45.4%), followed by femoral vein (40.2%) and the left internal jugular vein (14.4%). Baseline characteristics including comorbidities, history of medications, CRRT settings, and laboratory variables were similar between the two groups except for PT-INR levels which were less prolonged in patients with catheter dysfunction (1.5 ± 0.6 vs. 1.8 ± 1.1, *p* < 0.001). The mode of CRRT was continuous veno-venous hemodiafiltration for all cases included in analysis.

Multivariate analysis showed that less prolonged levels of PT-INR (adjusted odds ratio (OR) 0.49, 95% confidence interval (CI) 0.30–0.82,* p* = 0.007) and prolonged aPTT (OR 1.01, 95% CI 1.00–1.01, *p* = 0.049) were associated with increased the risk of catheter dysfunction (Table 2). Catheter tip location, mean blood flow rate of CRRT, and circuit anticoagulation were not associated with catheter dysfunction (Table 2). As an interaction between the catheter tip location and vascular access was identified, only the catheter tip location was included in the multivariate analysis.

**Table 2:** Generalized estimating equations with robust variance estimator analysis for catheter dysfunction

	Unadjusted OR (95% CI)	*p-*value	Adjusted OR (95% CI)	*p-*value
Age	1.02(1.00–1.04)	0.033	1.01(0.99–1.03)	0.163
Sex	1.09(0.67–1.78)	0.719		
Weight	1.00(0.99–1.02)	0.853		
Height	1.00(0.97–1.02)	0.707		
Body mass index	1.01(0.96–1.06)	0.654		
Hypertension	1.07(0.66–1.73)	0.788		
Diabetes mellitus	1.11(0.69–1.78)	0.672		
Dyslipidemia	1.21(0.75–1.96)	0.441		
Atrial fibrillation	0.72(0.31–1.66)	0.440		
Myocardial infarction	0.99(0.54–1.83)	0.979		
Stroke	2.19(1.06–4.51)	0.035	1.97(0.93–4.13)	0.078
Chronic kidney disease	1.20(0.73–1.98)	0.461		
Chronic hemodialysis	1.53(0.86–2.72)	0.146		
History of any thrombosis event	0.70(0.29–1.72)	0.437		
Medication of antiplatelet	0.84(0.45–1.57)	0.580	0.66(0.33–1.33)	0.248
Medication of anticoagulant	0.92(0.44–1.92)	0.834	1.02(0.55–1.92)	0.942
APACHE II score	1.01(0.98–1.04)	0.706		
Catheterized vessel				
Right internal jugular vein	Reference			
Left internal jugular vein	0.92(0.44–1.92)	0.834		
Femoral vein	1.04(0.63–1.72)	0.866		
Concurrent catheter in catheterized vessel	1.29(0.80–2.06)	0.295	1.80(0.95–3.39)	0.071
Concurrent catheter tip	1.01(0.62–1.64)	0.977	0.70(0.40–1.23)	0.828
Catheter tip location				
Superior vena cava	Reference		Reference	
Right atrium	0.28(0.08–0.94)	0.039	0.31(0.09–1.12)	0.074
Left innominate vein	1.14(0.44–2.92)	0.790	1.32(0.46–3.74)	0.604
Inferior vena cava	1.01(0.21–4.85)	0.990	1.36(0.25–7.35)	0.722
Iliac vein	0.88(0.53–1.45)	0.604	1.02(0.56–1.86)	0.953
Mean blood flow	1.00(0.99–1.01)	0.440	1.00(0.99–1.00)	0.680
Circuit anticoagulation	1.26(0.76–2.10)	0.371	1.06(0.61–1.87)	0.828
Causes of CRRT				
End stage renal disease	Reference			
Sepsis	0.85(0.44–1.65)	0.627		
Nephrotoxic kidney injury	0.61(0.22–1.72)	0.350		
Ischemic kidney injury	0.85(0.44–1.61)	0.614		
Others	0.75(0.28–2.03)	0.577		
Use of vasopressor	0.76(0.45–1.29)	0.311		
Platelet count	1.00(1.00–1.00)	0.229		
Prothrombin time	0.56(0.36–0.89)	0.013	0.49(0.30–0.82)	0.007
aPTT	1.00(0.99–1.01)	0.547	1.01(1.00–1.01)	0.049
hs-CRP level	0.99(0.97–1.02)	0.512		

*Abbreviations*: *OR* odds ratio, *CI* confidence interval, *APACHE* acute physiology and chronic health evaluation, *CRRT* continuous renal replacement therapy, *aPTT* activated partial thromoplastin time, *hs-CRP* high sensitivity C-reactive protein

Regarding the association between bleeding events during vascular access and the levels of PT-INR and aPTT, there were no major bleeding events (requiring fluid resuscitation, transfusion, or mechanical hemostasis) 9 minor bleeding events (hematoma formation or blood oozing at the catheter insertion sites) among 507 catheterizations. There was no statistically significant difference in PT-INR (1.7 ± 0.8 vs. 1.8 ± 1.0, *p* = 0.380) or in aPTT levels (62.6 ± 54.7 vs. 52.8 ± 32.4, *p* = 0.928) when comparing cases with bleeding events to those without bleeding events.

Compared to catheters placed in right internal jugular veins, the risk of re-catheterization was higher when catheters were placed in the left jugular vein (adjusted OR 9.92, 95% CI 2.71–36.36, *p* < 0.001), or the femoral vein (adjusted OR 4.33, 95% CI 1.27–14.76, *p* = 0.019). Patients with history of myocardial infarction showed lower risk for re-catheterization (adjusted OR 0.11, 95% CI 0.02–0.77, *p* = 0.026) and of PT-INR or aPTT levels were not associated with re-catheterization (Table 3).

**Table 3:** Generalized estimating equations with robust variance estimator analysis for re-catheterization

	Unadjusted OR (95% CI)	*p-*value	Adjusted OR (95% CI)	*p-*value
Age	1.03(0.99–1.06)	0.122	1.03(1.00–1.07)	0.075
Sex	1.40(0.67–2.91)	0.372		
Weight	1.03(0.99–1.06)	0.296		
Height	0.98(0.94–1.03)	0.432		
Body mass index	0.97(0.89–1.06)	0.545		
Hypertension	0.90(0.43–1.89)	0.779		
Diabetes mellitus	0.70(0.32–1.53)	0.367		
Dyslipidemia	0.80(0.36–1.81)	0.597		
Atrial fibrillation	1.38(0.45–4.26)	0.575		
Myocardial infarction	0.15(0.02–1.07)	0.058	0.11(0.02–0.77)	0.026
Stroke	-	-		
Chronic kidney disease	1.05(0.47–2.35)	0.910		
Chronic hemodialysis	0.68(0.24–1.98)	0.483		
History of any thrombosis event	0.28(0.04–2.04)	0.210		
Medication of antiplatelet	0.67(0.23–1.96)	0.465	0.98(0.30–3.23)	0.974
Medication of anticoagulant	0.61(0.21–1.77)	0.358	0.56(0.18–1.77)	0.323
APACHE II score	1.04(0.99–1.08)	0.124		
Catheterized vessel				
Right internal jugular vein	References			
Left internal jugular vein	7.00(2.03–24.15)	0.002	9.92(2.71–36.36)	< 0.001
Femoral vein	5.81(1.95–17.31)	0.002	4.33(1.27–14.76)	0.019
Concurrent catheter in catheterized vessel	0.40(0.17–0.97)	0.043	0.44(0.11–1.81)	0.255
Concurrent catheter tip	0.85(0.41–1.76)	0.668	1.07(0.5–2.33)	0.857
Catheter tip location	-			
Mean blood flow	1.01(0.99–1.02)	0.314	1.01(0.99–1.02)	0.385
Circuit anticoagulation	1.46(0.66–3.20)	0.346	1.32(0.55–3.17)	0.529
Causes of CRRT				
End stage renal disease	Reference			
Sepsis	1.56(0.58–4.15)	0.375		
Nephrotoxic kidney injury	1.64(0.44–6.11)	0.461		
Ischemic kidney injury	0.47(0.14–1.55)	0.215		
Others	0.38(0.05–3.11)	0.366		
Use of vasopressor	1.40(0.55–3.52)	0.480		
Platelet count	1.00(0.99–1.00)	0.415		
Prothrombin time	0.71(0.37–1.39)	0.320	0.82(0.39–1.73)	0.609
aPTT	0.99(0.98–1.01)	0.298	1.00(0.99–1.01)	0.525
hs-CRP level	1.01(0.97–1.05)	0.642		

*Abbreviations*: *OR* odds ratio, *CI* confidence interval, *APACHE* acute physiology and chronic health evaluation, *CRRT* continuous renal replacement therapy, *aPTT* activated partial thromoplastin time, *hs-CRP* high sensitivity C-reactive protein

**Fig. 2:**
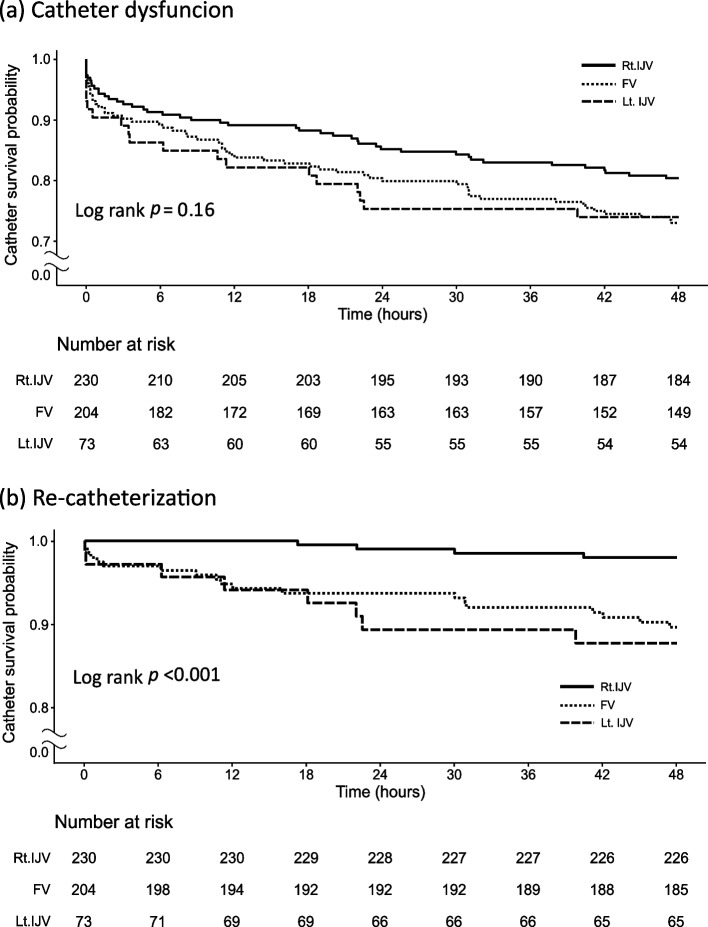
Kaplan–Meier survival curve of catheter after catheterization. (**a**) catheter dysfunction, and (**b**) re-catheterization

Figure 2 showed the timing of catheter dysfunction and re-catheterization within 48 h from initiation of CRRT according to the site of vascular access. More than 50% of catheter dysfunctions occurred in the first 6 h after CRRT initiation. The overall catheter survival probability in the right internal jugular vein seemed to be higher compared to other sites but did not reach statistical significance (Fig. 2a). Time to re-catheterization was longest for the right jugular vein compared to left internal jugular or femoral vein (Fig. 2b). There was no difference in catheter survival between the femoral vein and the left internal jugular vein (*p* = 0.68).

## Discussion

In this retrospective study, less prolonged levels of PT and slightly prolonged aPTT were identified as risk factors for catheter dysfunction during the first 48 h after initiation of CRRT. In terms of requirement of re-catheterization to maintain CRRT, venous access site was associated with the increased risk of re-catheterization. Initial catheterization in the left jugular vein or femoral vein as venous access sites were more likely to require re-catheterization compared to the right internal jugular vein.

Previous studies of risk factors associated with catheter dysfunction during RRT have focused on the vascular access site with conflicting reports with respect to relative risk of dysfunction between catheters in the femoral and the internal jugular vein. The internal jugular vein had been considered as the optimal site of vascular access for RRT with lower incidence of catheter dysfunction and risk of catheter-related infection [23–25]. However, since the CATHEDIA study, a multicenter randomized controlled trial of patients undergoing intermittent hemodialysis or CRRT, there have been reports of similar risk of infection, catheter dysfunction, and dialysis performance between femoral and right jugular vein catheterization [17, 26, 27]. Recent guidelines recommend both femoral and right jugular veins as the initial site for vascular access in the setting of critical care, while discouraging the use of left jugular and subclavian veins [8, 28]. In clinical practice, femoral vein is preferred for CRRT especially for sicker and lighter patients [11, 18], as it enables faster and more successful vascular access [27].

Type, length, and the location of tip of catheter have also been shown to be associated with the risk of catheter dysfunction. The non-tunneled, non-cuffed temporary catheter is recommended for critically ill patient as the catheterization procedure at bedside is straightforward and does not require fluoroscopic guidance [29–31]. However, non-tunneled catheters are associated with higher rates of dialysis interruption, lower venous and arterial access pressure, higher incidence of re-catheterization, and decreased filter life during CRRT [19, 32]. As for the length of the catheter, catheters placed in the femoral vein are usually longer [11, 33] as it is associated with less catheter dysfunction [17]. The catheter tip location is also widely recognized to be related to catheter dysfunction, with positioning it in the right atrium or inferior vena cava known to reduce the risk of dysfunction [14, 33]. In this study, however, there was no significant association between the catheter tip location and catheter dysfunction (Table 2). We suggested that catheter dysfunction in the early period following CRRT initiation may be primarily influenced by factors such as vascular access or laboratory variables, rather than the catheter tip location.

Risk factors of catheter dysfunction other than the vascular access remain unclear in patients undergoing CRRT since most studies evaluated patients requiring iHD. In a prospective observational study that analyzed clinical and laboratory variables in addition to vascular access sites for the risk of non-tunneled non-cuffed catheter malfunction during iHD, femoral vein access was identified as the sole independent risk factor [25]. In a retrospective study analyzing potential risk factors of tunneled hemodialysis catheter dysfunction, female sex, normal PT levels, and left internal jugular vein access were associated with catheter dysfunction [14]. A more recent competing risk analysis study evaluating potential predictors for thrombosis and infection of tunneled hemodialysis catheters showed similar results with the aforementioned study. Female sex showed a near two-fold risk of catheter thrombosis compared to male sex and hypertension and obesity were also identified as risk factors [15]. Several prospective studies have shown higher risk of catheter thrombosis in females compared to males [16, 34], which is contrary to risk of venous thrombosis with regards to sex [35, 36]. Relative size discrepancy between the catheter and the accessed vein, or location of catheter tip, which both directly affect the flow rate, may have contributed to the increased risk of catheter dysfunction in females [37–39]. Hypertension has been reported as a risk factor in another single-center study to increase the risk of catheter dysfunction, not only as a cause of hemodialysis but also as a comorbidity [40].

The association between less prolonged levels of PT and increased risk of catheter dysfunction requires careful and cautious interpretation. As the levels of PT in patients with catheter dysfunction were also higher than the normal range, it would be more appropriate to interpret the result that the higher level of PT decreased the risk of catheter dysfunction, rather than the lower level of PT increased the risk. Regarding the levels of aPTT, which were slightly prolonged in patients with catheter dysfunction, we found that the prolongation was primarily attributable to the patients requiring switch of lumen. The aPTT levels in patients requiring catheter repositioning or re-catheterization were relatively lower compared to those requiring a lumen switch, and even among patients without catheter dysfunction, although the difference did not reach statistical significance. In addition to the use of intra-circuit nafamostat mesilate to reduce circuit thrombosis, it may be possible that anticoagulation using low-molecular-weight heparin in this study may have influenced the aPTT levels. However, considering the impact of PT levels, data seems to be insufficient to provide a plausible explanation for the association of prolonged aPTT with catheter dysfunction.

Vascular access site was significantly associated with severe catheter dysfunction that requires re-catheterization during the early period of CRRT. Left jugular and femoral access were significantly associated with increased risk of re-catheterization compared to the right jugular access, after adjusting for relevant variables including body mass index. Considering that re-catheterization was required in 10 out of 31 patients within 6 h after catheter insertion, it is more likely that the vascular access site (as opposed to catheter tip thrombosis) may influence the optimal catheter location where adequate flow through the catheter can be generated. Concurrent catheter placement in the same vascular access or the catheter tip location were not associated with catheter dysfunction or re-catheterization in our study, suggesting that disruption of blood flow may have occurred within the catheter, rather than the catheter orifice. The long and angulated path of the catheter through the left jugular access or kinking of catheter through the femoral access with the patient in the sitting position may potentially impede blood flow through the catheter despite that the location of the catheter tip is adequate.

As previous studies have focused on vascular access and other risk factors were only studied in out-patient clinic patients undergoing iHD with a longer follow-up duration, our study aimed to evaluate potential risk factors including comorbidities, previous or concurrent anticoagulation, and CRRT variables such as blood flow rate or intra-circuit anticoagulation. This study also focused on early catheter dysfunction, especially during the first 48 h following initiation of CRRT, as maintenance of stable RRT is crucial for critically ill patients, especially with severe metabolic acidosis or electrolyte imbalance [11–13]. By defining the need for switching catheter limbs, catheter reposition, and re-catheterization as catheter dysfunction, all potential risk factors interrupting adequate blood flow through the circuit were analyzed. For patients requiring re-catheterization, subgroup analysis was conducted to investigate risk factors causing catheter malfunction, resulting in unintended interruption of CRRT.

This study has several limitations that should be considered. First, due to the retrospective nature of the study, catheter dysfunction was defined based on interventions executed to improve blood flow through the catheter. The cause of catheter dysfunction was also not assessed. However, re-catheterization due to infection were excluded as the risk of catheter associated infection was relatively low during the first 48 h after catheter insertion. To reduce the potential influence of pre-existing venous thrombosis, only one catheterization per vascular access was included during the study period. Second, there were some variations as to the physicians performing the catheterization and the proficiency of the operators was not assessed. However, all catheterizations were performed either by experienced board-certified intensivists or residents under supervision of the intensivists. In addition, all catheterizations were performed with real-time ultrasound guidance following standardized technique and protocol.

In conclusion, less prolonged (close to normal) levels of PT was associated with catheter dysfunction within 48 h after initiation of CRRT. Left jugular and femoral vascular access were associated with catheter dysfunctions that required re-catheterization.

## Data Availability

The dataset used and analyzed during the current study are available from the corresponding author on reasonable request.

## Abbreviations

APACHE II: Acute physiology and chronic health evaluation II
aPTT: Activated partial thromboplastin time
CRRT: Continuous renal replacement therapy
hs-CRP: High-sensitivity C-reactive protein
ICU: Intensive care units
iHD: Intermittent hemodialysis
PT-INR: Prothrombin time international normalized ratio
RRT: Renal replacement therapy
